# Impact of miglustat on evolution of atypical presentation of late-infantile-onset Niemann–Pick disease type C with early cognitive impairment, behavioral dysfunction, epilepsy, ophthalmoplegia, and cerebellar involvement: a case report

**DOI:** 10.1186/s13256-016-1038-9

**Published:** 2016-09-06

**Authors:** Jean-Marie Cuisset, S. Sukno, A. Trauffler, P. Latour, D. Dobbelaere, L. Michaud, L. Vallée

**Affiliations:** 1 Service de Neuropédiatrie, Hôpital Roger Salengro, Lille, France; 2 Unité de Neuropédiatrie, Hôpital Saint Vincent, Lille, France; 3 Service de Neurobiologie, CHU de Lyon – GH Est, Bron, France; 4 Centre de référence de Lille des Maladies Héréditaires du Métabolisme, Hôpital Jeanne de Flandre, Lille, France; 5 Service de Gastropédiatrie, Hôpital Jeanne de Flandre, Lille, France; 6 Hôpital Roger Salengro, CHRU, Boulevard du Pr Jules Leclercq, 59037 CEDEX, Lille France

**Keywords:** Niemann–pick disease type C, Late-infantile, Cognition, Epilepsy, Miglustat

## Abstract

**Background:**

Niemann–Pick disease type C is a rare inherited neurodegenerative disease involving impaired intracellular lipid trafficking and accumulation of glycolipids in various tissues, including the brain. Miglustat, a reversible inhibitor of glucosylceramide synthase, has been shown to be effective in the treatment of progressive neurological manifestations in pediatric and adult patients with Niemann–Pick disease type C, and has been used in that indication in Europe since 2010.

**Case presentation:**

We describe the case of a 16-year-old white French boy with late-infantile-onset Niemann–Pick disease type C who had the unusual presentation of early-onset behavioral disturbance and learning difficulties (aged 5) alongside epileptic seizures. Over time he developed characteristic, progressive vertical ophthalmoplegia, ataxic gait, and cerebellar syndrome; at age 10 he was diagnosed as having Niemann–Pick disease type C based on filipin staining and genetic analysis (heterozygous I1061T/R934X *NPC1* mutations). He was commenced on miglustat therapy aged 11 and over the course of approximately 3 years he showed a global improvement as well as improved cognitive and ambulatory function. During this period he remained seizure free on antiepileptic therapy, using valproate and lamotrigine.

**Conclusions:**

Miglustat improved the neurological status of our patient, including seizure control. Based on our findings in this patient and previous published data, we discuss the importance of effective seizure control in neurological improvement in Niemann–Pick disease type C, and the relevance of cerebellar involvement as a possible link between these clinical phenomena. Thus the therapeutic efficacy of miglustat could be hypothesized as a substrate reduction effect on Purkinje cells.

## Background

Niemann–Pick disease type C (NP-C) is a rare neurodegenerative disease caused by autosomal recessive mutations in either the *NPC1* gene (in 95 % of cases) or the *NPC2* gene [1], resulting in impaired intracellular lipid trafficking and accumulation of glycolipids in various tissues including the brain [2]. The diagnosis incidence of NP-C has been reported as 1 case in every 120,000 live births [1].

In clinical terms NP-C is characterized by a range of symptoms including progressive neurological manifestations, cognitive and behavioral impairment, and systemic symptoms [1]. The severity and rate of progression of NP-C varies with age at neurological disease onset [3]. For this reason it is helpful to regard different clinical forms of the disease according to agreed age-at-onset categories: early-infantile (at age 3 months to <2 years), late-infantile (at age 2 to <6 years), juvenile (at age 6 to 15 years), and adolescent/adult (at age ≥15 years) [1].

Behavioral problems, impaired learning, and delayed or arrested speech development are usually observed in patients with neurological disease onset from the juvenile period onwards [1, 2]. Cognitive impairment with or without psychiatric manifestations usually only appears after a prolonged period of disease progression, and is most often recognized in adolescent/adult-onset patients [2, 4].

Epileptic seizures of any type (for example, partial or generalized, myoclonic, tonic–clonic) and cataplexy are most often seen in patients with late-infantile-onset NP-C [1]. While seizures vary markedly among patients in terms of their intensity and frequency, seizure activity represents an important clinical management issue in NP-C because it can have a major impact on a patient’s quality of life and, in some cases, prognosis [1, 5].

We describe the case of a boy with late-infantile-onset NP-C who had the unusual presentation of early-onset behavioral disturbance and learning difficulties; he later developed epileptic seizures as well as overt neurological manifestations of NP-C including cerebellar signs and ophthalmoplegia.

## Case presentation

We describe the case of a 16-year-old white French boy born to unaffected parents in late-1999 following a normal pregnancy and birth. He displayed normal psychomotor development up to 4 years of age, with autonomous walking at 13 months and speech in which he used words and sentences at 18 months.

At 5 years of age he began to show cognitive slowing and graphic difficulties (poor drawing) in kindergarten, as well as motor dyspraxia. Over the next 1.5 years his motor function deteriorated further with the development of cerebellar syndrome, and he began to experience partial seizures primarily affecting his right frontal lobe. These seizures were treated initially using oxcarbazepine, which was replaced after 5 months by lamotrigine due to the appearance of myoclonia. Laboratory analyses for a panel of metabolic diseases revealed normal urine ammonia levels, normal plasma acylcarnitine, normal leukocyte acid hydrolase activities (β-D-galactosidase, hexosaminidases A and B, arylsulfatase A, β-D-mannosidase), and normal copper levels. Chromatographic urine analysis showed normal levels of organic acids and oligosaccharides. G-band karyotype analysis showed normal findings, and an *FMR1* gene study ruled out fragile X syndrome.

Follow up at 9 years of age showed further cognitive deterioration, categorized as a frontal syndrome (echolalia, behavioral perseveration) and progressive loss of expressive language. His cerebellar syndrome had worsened, with occurrence of an ataxic gait and dysmetria, and he displayed pyramidal syndrome. He was presenting several epileptic seizures daily. He had also begun to be incontinent for urine and feces.

**Fig. 1:**
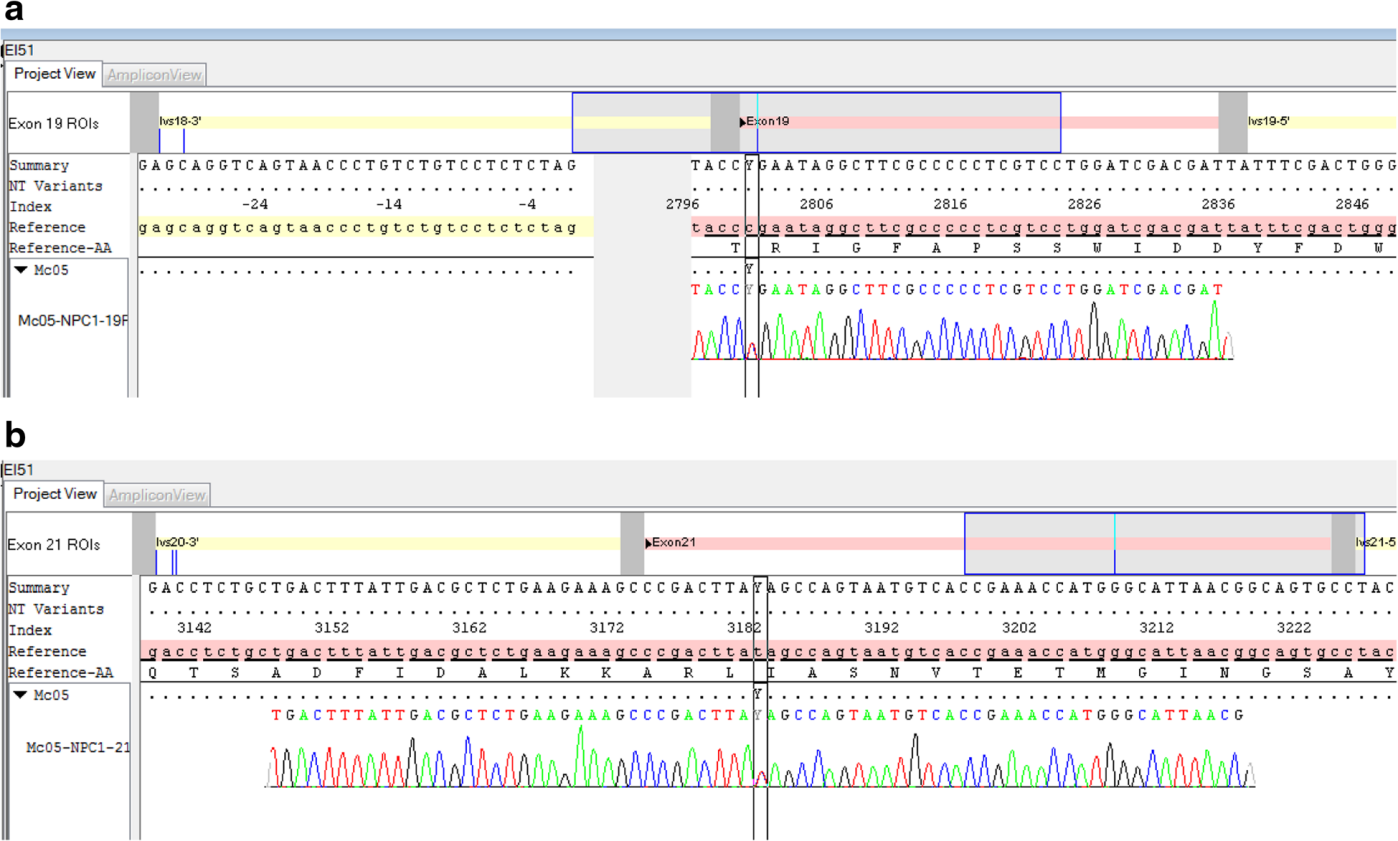
**a** *NPC1* gene – exon 19: mutation c.2800C>T heterozygous (p.Arg 934*). **b** *NPC1* gene – exon 21: mutation c.3182T>C heterozygous (p.Ile1061Thr)

Brain magnetic resonance imaging (MRI) did not identify any abnormalities, but an ophthalmic examination revealed vertical supranuclear gaze palsy (VSGP), and analysis of peripheral blood and bone marrow biopsies identified vacuolated histiocytes. Together, these findings prompted biochemical and genetic testing for suspected NP-C in early 2010, which was approximately 5 years before this report. Filipin staining in cultured skin fibroblasts identified cholesterol-rich perinuclear vesicles. Plasma oxysterols (cholestan-3β,5α,6β-triol) have not been tested for this patient. Sequencing of the complete coding sequences and intronic junctions of *NPC1* and *NPC2* genes was done by Sanger method on genomic deoxyribonucleic acid (DNA) extracted from his fibroblasts (used for the filipin test). Two deleterious heterozygous mutations in *NPC1* (reference transcript NM_000271.4) were found: p.Arg934* (c.2800C>T) in exon 19 (Fig. 1a) and p.Ile1061Thr (c.3182T>C) in exon 21 (Fig. 1b). The independent segregation of the two mutations was demonstrated by studying his parents’ DNA obtained from blood samples.

So, he had a typical compound heterozygote NP-C genotype: I1061T/R934X.

Soon after diagnosis he was hospitalized in early 2010 following two episodes of aspiration-related pneumonia. A speech therapist examination identified frequent aspiration of liquids, sialorrhea/frequent drooling, a weakened gag reflex, and slow swallowing and ingestion of solid food. A gastrostomy tube was placed approximately 1 month later. He scored 9/9 (restricted to a wheelchair) on the Hauser Standard Ambulation Index (SAI), 17 out of a possible maximal severity score of 18 on the NP-C disability scale proposed by Iturriaga *et al*. [6], and a maximal severity score of 1.0 on the modified disability scale reported by Pineda *et al*. [7]. A follow-up ophthalmological evaluation was not possible as our patient had complete ophthalmoplegia. His brain MRI findings were normal.

Treatment with miglustat (Zavesca®; Actelion Pharmaceuticals Ltd) was commenced in February 2010, and he received approximately 2.5 years of uninterrupted therapy at a dose of 100 mg three times a day based on his body surface area (as per manufacturer’s recommendations) [8]. At the commencement of therapy he experienced initial, transient abdominal pain and frequent diarrhea that were treated using loperamide 1 mg twice a day. He was initiated on a diet excluding disaccharides following consultation with a specialist dietician, and his feeding and caloric intake have since been monitored regularly by a gastropediatrician.

Electroencephalographic evaluation indicated infraclinical brain function abnormalities (spike waves) predominantly affecting his left temporal lobe. Motor and sensory nerve conduction velocities were normal. His epilepsy appeared to be well controlled in late-2010. He was at that time receiving a combination of sodium valproate (400 mg three times a day; 30 mg/kg/day) and lamotrigine (100 mg in the morning and 50 mg in the evening; 4 mg/kg/day for 16 months). He became seizure free in June 2010, 4 months after beginning the miglustat treatment.

**Fig. 2:**
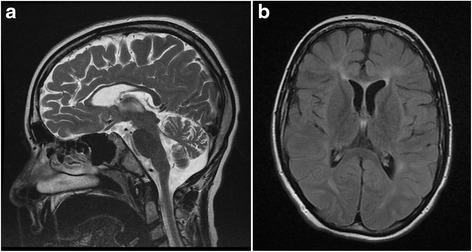
Magnetic resonance imaging scan from patient with late-infantile-onset Niemann–Pick disease type C. The magnetic resonance imaging scan shows: **a** vermian atrophy on T2-weighted sequence, May 2012 and; **b** diffuse white matter hyperintensities on fluid-attenuated inversion recovery sequence (FLAIR), May 2012

At clinical assessment in May 2012 (approximately 3 years before this presentation), at 12.5 years of age, following approximately 2.5 years of miglustat therapy, a global improvement was recorded. His body weight had increased and he had not experienced an epileptic seizure for approximately 2 years (since May 2010), which seemed to coincide with the time at which good digestive tolerance to miglustat therapy was achieved. He showed improvements in cognition (reacquisition of a few words) and motor function, and was ambulating with a walker, scoring 5 on the SAI: he was able to walk 7.62 m (25 feet) in <20 seconds using a walker. He displayed ataxic, stop-start ambulation, dysmetria, and upper limb apraxia; his ophthalmoplegia remained unchanged. A repeat brain MRI examination showed bilateral atrophy of his cerebellar vermis and diffuse white matter hyperintensities in T2-weighted images (Fig. 2a, b). He was therefore scheduled for full clinical assessment in line with international clinical recommendations in order to reassess his miglustat dose based on a judgment of efficacy versus safety.

At further follow up he scored 14 on the Iturriaga *et al*. NP-C disability scale [6], and 0.7 on the modified disability scale published by Pineda *et al*. [7]. Since his body weight had reached 40 kg, corresponding to a body surface area of 1.28 m^2^, the miglustat dose was increased from 100 mg three times a day to 200 mg three times a day according to the French recommendations for the use of miglustat in children with NP-C.

## Discussion

In NP-C, mutations in the *NPC1* gene give rise to impaired intracellular lipid trafficking and subsequent accumulation of unesterified cholesterol, sphingosine, and a range of glycosphingolipids in various tissues including the brain [2]. The cerebellum plays an important role in NP-C, as neuronal loss is more prominent compared with other brain areas, and outputs from the cerebellum operate in both the motor and cognitive domains [9]. GABAergic cerebellar Purkinje cells appear particularly susceptible to damage in NP-C. Based on data from a feline NP-C model, Stein *et al*. proposed that miglustat – a reversible inhibitor of glucosylceramide synthase, the enzyme that catalyzes the first step in the biosynthesis of most glycolipids in NP-C – might improve neurological function due partly to improved Purkinje cell survival [10].

Learning disabilities observed in the nursery school setting can be the first manifestations of the late-infantile form of NP-C, as observed in our patient. However, this case was slightly unusual in that patients with NP-C do not generally present with cognitive impairment and/or behavioral problems so early in life, certainly not during the late-infantile period [1, 2]. Cognitive deterioration is usually first observed during adolescence or early-adulthood [1, 2, 4].

This case also highlights the importance of achieving effective control of epilepsy in patients with NP-C. Seizures can have a notable impact on a patient’s quality of life and general health status, and usually result in worsening patient scores on the NP-C disability scale, which assesses four key parameters of neurological disease progression: ambulation, manipulation, language, and swallowing [1, 6, 7]. In fact, patients with frequent and/or severe seizures are considered to have a poor overall prognosis [1].

Skorpen *et al*. reported the case of a patient with p.S940L-homozygous late-infantile-onset NP-C whose quality of life and neurologic condition initially improved with miglustat [5]. However, follow up 18 months after commencing miglustat therapy, at which point the patient was experiencing very frequent epileptic attacks, indicated a general loss of energy and deterioration of neurological status, and a worsening of scores on the modified disability scale from 0.63 to 0.90 at the peak of epileptic activity. When control of seizure epilepsy was re-established 4 months later, a repeat assessment indicated a return toward neurological stability (disability scale score 0.71) [5]. Based on these observations the authors emphasized the importance of seizure control therapy in achieving and maintaining neurological stabilization.

Similar findings were reported based on a prospective study of miglustat therapy in a French NP-C cohort, which included eight patients with the early-infantile form, eight with the late-infantile form, and three with the juvenile-onset form of the disease (our patient was included as patient 17 in this overall cohort) [11]. While neurological deterioration was seen to commence in one patient at the time his seizures became refractory to antiepileptic medication, pre-existing epilepsy (before miglustat therapy) or the onset of epilepsy during miglustat therapy did not appear to affect neurological outcomes in cases where seizure control was maintained using antiepileptic medications [11]. In our patient, the antiepileptic therapy using valproate and lamotrigine showed no efficacy for more than 1 year; however, our patient’s condition dramatically improved after miglustat therapy was started and he became seizure free 4 months after its introduction. This chronology of events seems to be suggestive of the efficacy of miglustat rather than the antiepileptic therapy alone.

Miglustat has been shown to stabilize neurological manifestations in children with NP-C, particularly among those with late-infantile or juvenile-onset disease [7, 11]. Our patient, who has received uninterrupted treatment with miglustat for approximately 3 years, showed improved neurological status (reacquisition of verbal speech and indoor assisted ambulation) after 5 months on miglustat therapy, and has remained free of seizure activity. He remains neurologically stable to date, despite showing evidence of vermian atrophy and diffuse white matter hyperintensities in T2-weighted images during a brain MRI assessment in May 2012 (after 15 months of miglustat therapy). This seems consistent with previous data from longitudinal imaging studies in pediatric patients with NP-C, which indicated no clear relationship between MRI or magnetic resonance spectroscopy (MRS) findings and clinical disease course during miglustat therapy [11–13].

On the basis of this case, it is interesting to speculate whether early-onset behavioral/cognitive deterioration may be partly related to seizure activity in NP-C. Further, it might be pertinent to investigate whether the therapeutic effects of miglustat in NP-C might be related, at least in part, to the improved survival of neuronal cerebellar substrates associated with both cognitive function and seizures. In this respect, it seems relevant to note that cerebellar grey matter volume has been shown to be reduced in patients with generalized tonic–clonic seizures [14], and that there is evidence to suggest that altered cerebellar GABAergic neurotransmission can affect both seizure activity and cognitive/behavioral function [15].

## Conclusions

Miglustat in our patient with late-infantile-onset NP-C has improved neurological manifestations and may have contributed to improve his epileptic status, since seizure control occurred after its initiation. We hypothesize a common cerebellar implication in both cognitive/behavior disturbances and seizure activity, and thus a possible therapeutic effect of miglustat by a substrate reduction therapy on Purkinje cells. However, as suggested by Stein *et al*., miglustat could improve neurological manifestations of NP-C disease by other mechanisms, including reduced glycosphingolipid accumulation in the brain, and modulation of microglial immunophenotype and function [10].

## Abbreviations

NP-C: Niemann–Pick disease type C
MRI: Magnetic resonance imaging
VSGP: Vertical supranuclear gaze palsy
SAI: Standard Ambulation Index
MRS: Magnetic resonance spectroscopy
